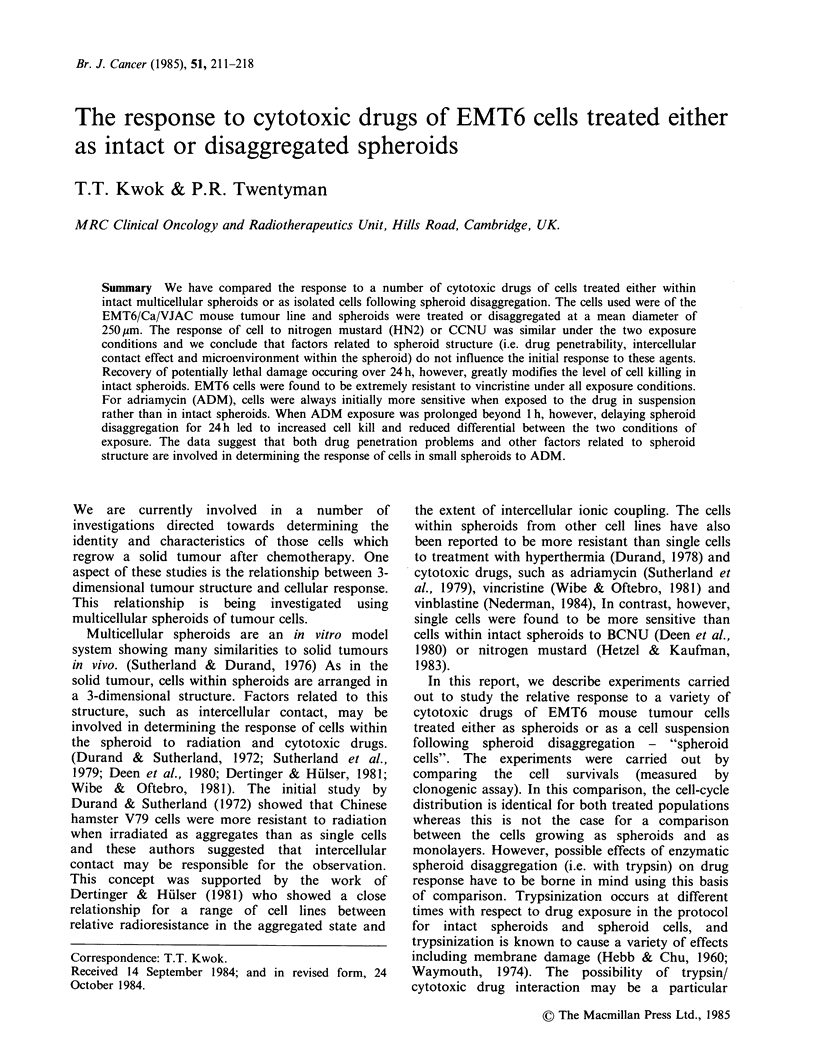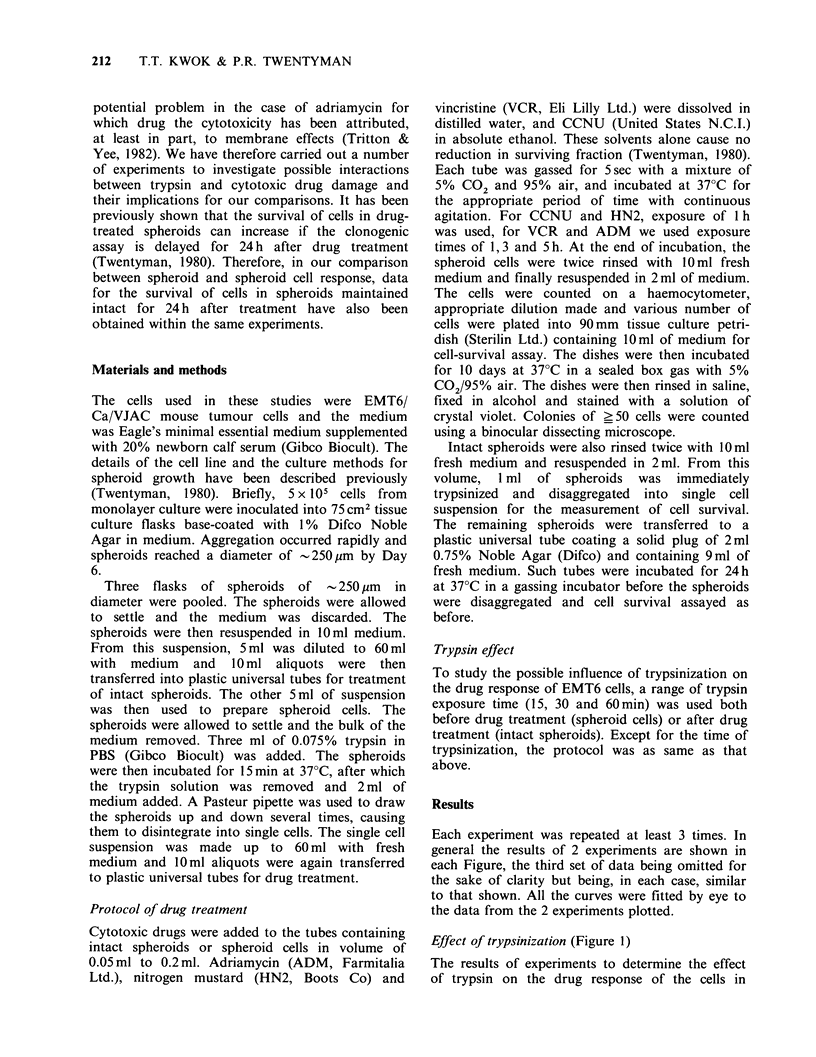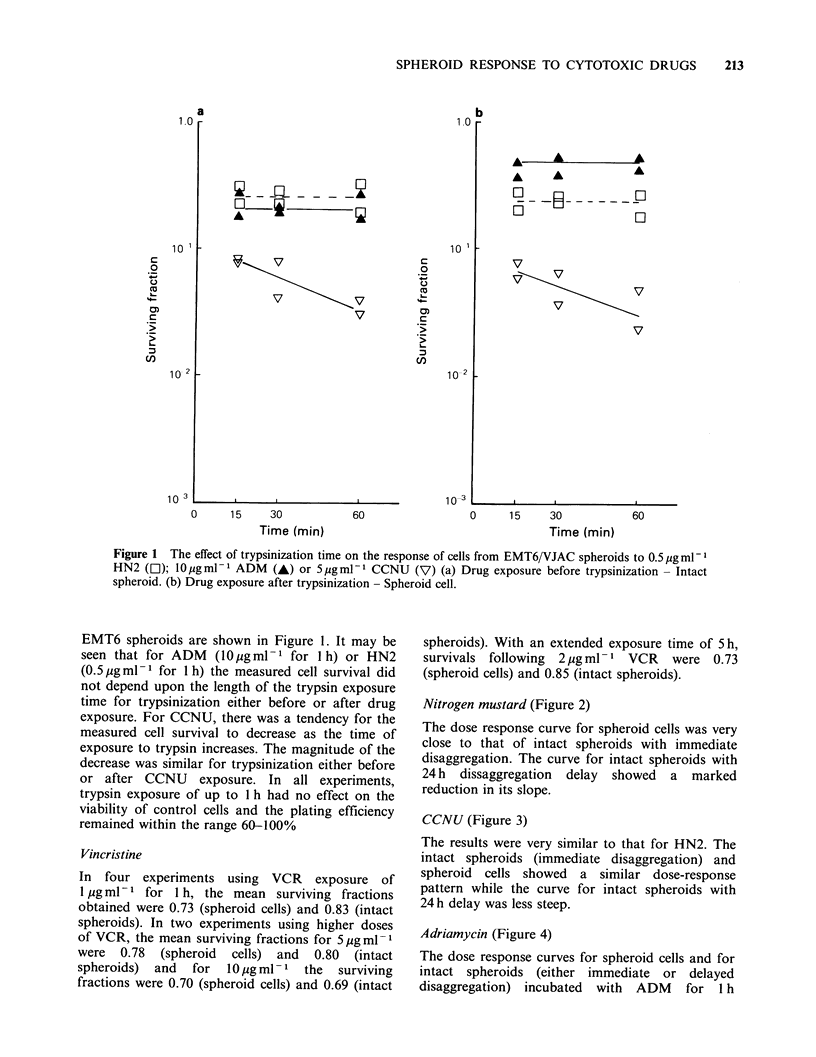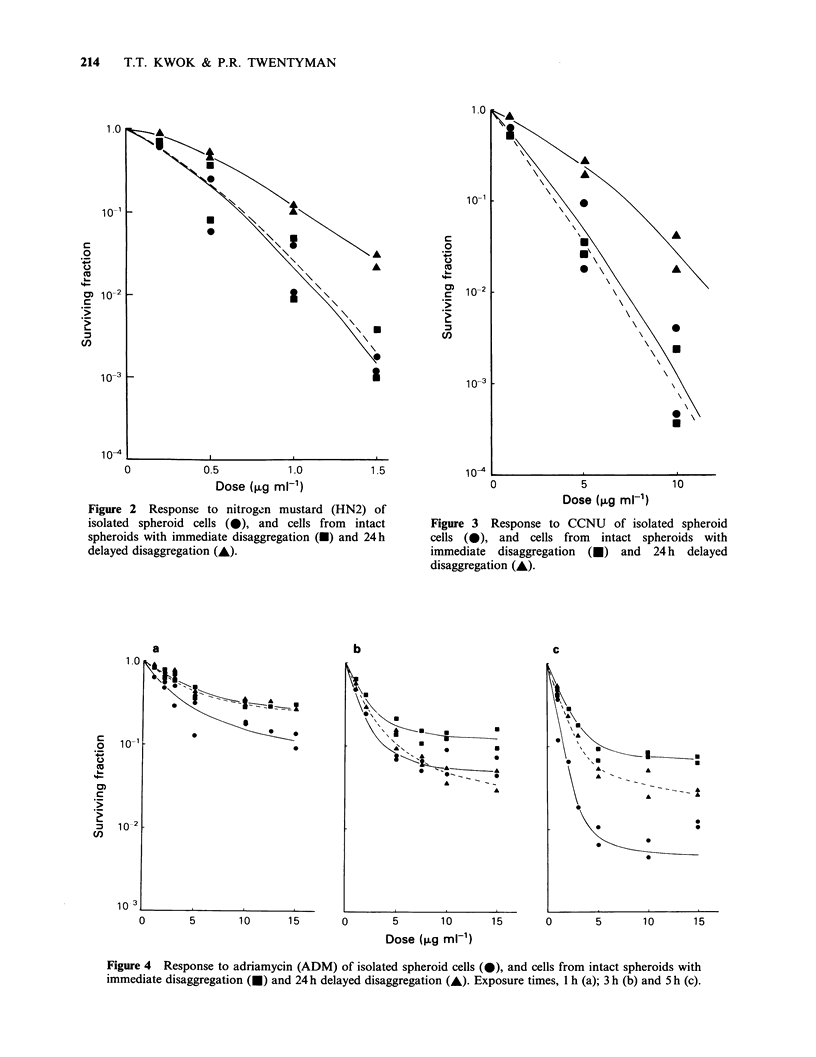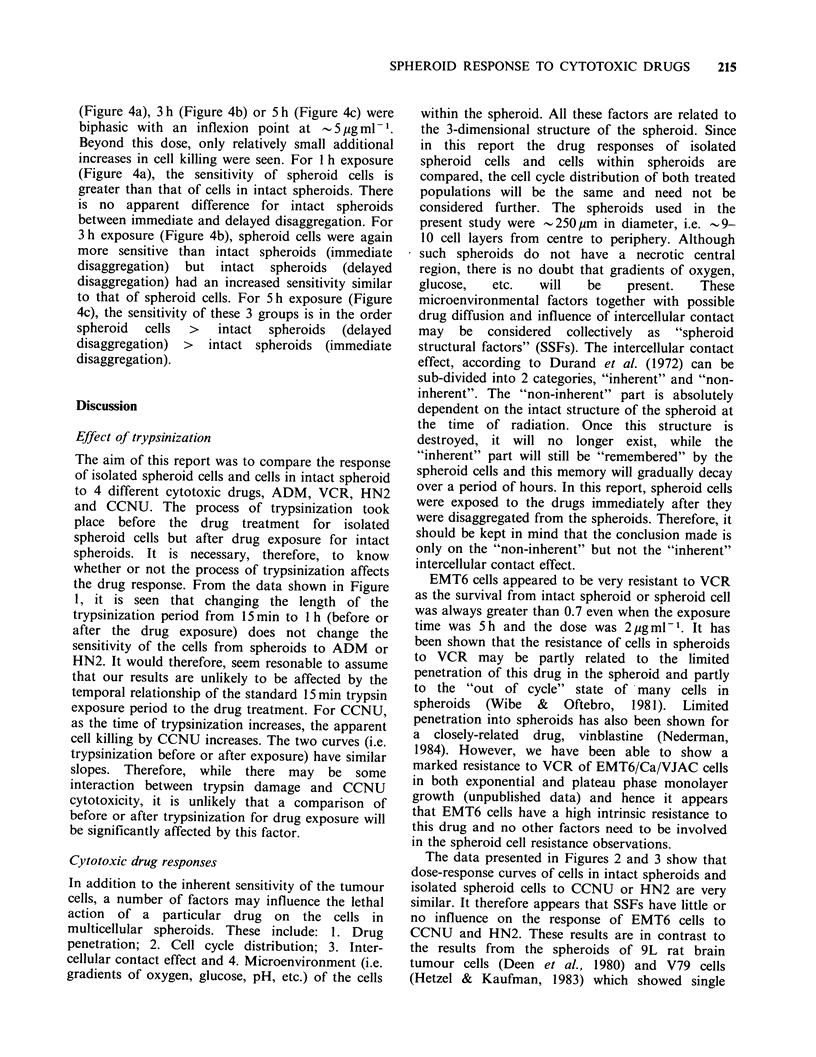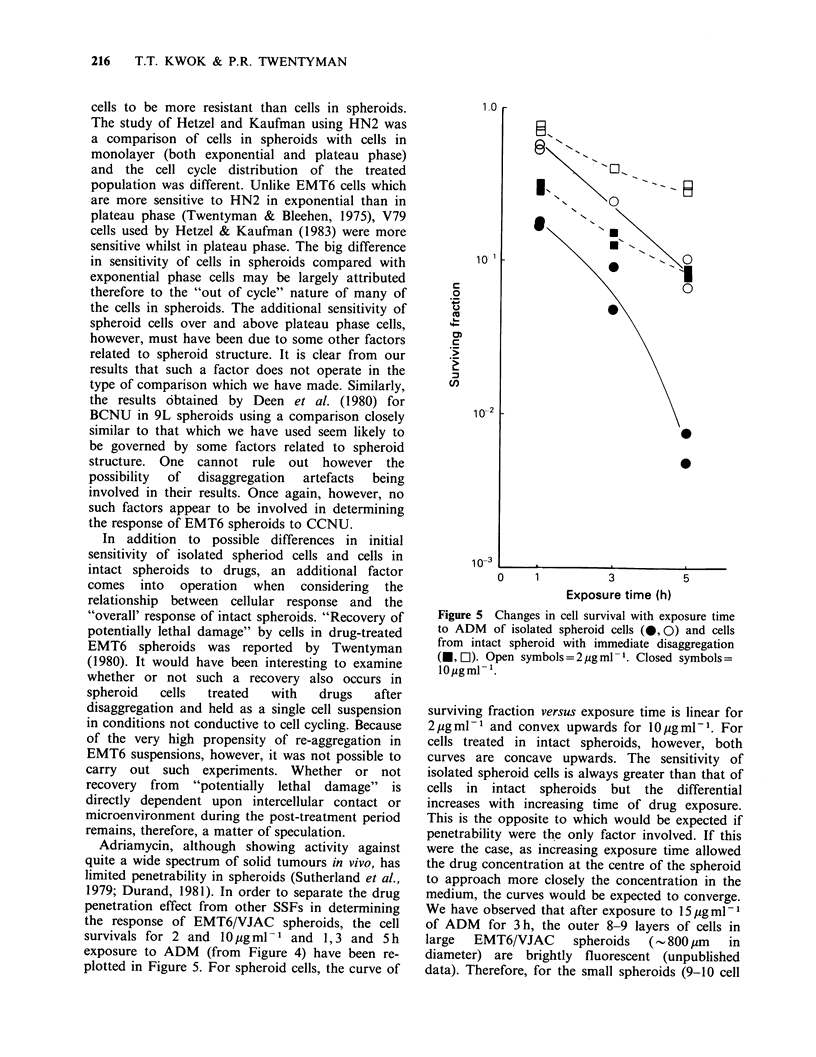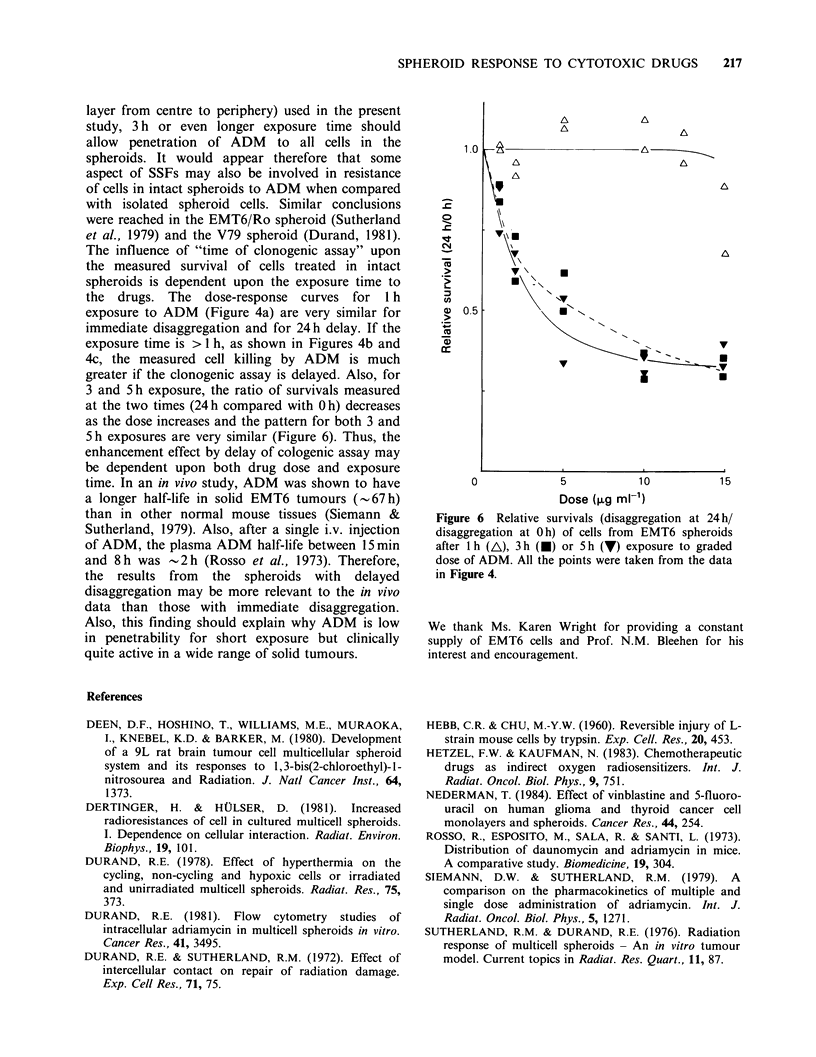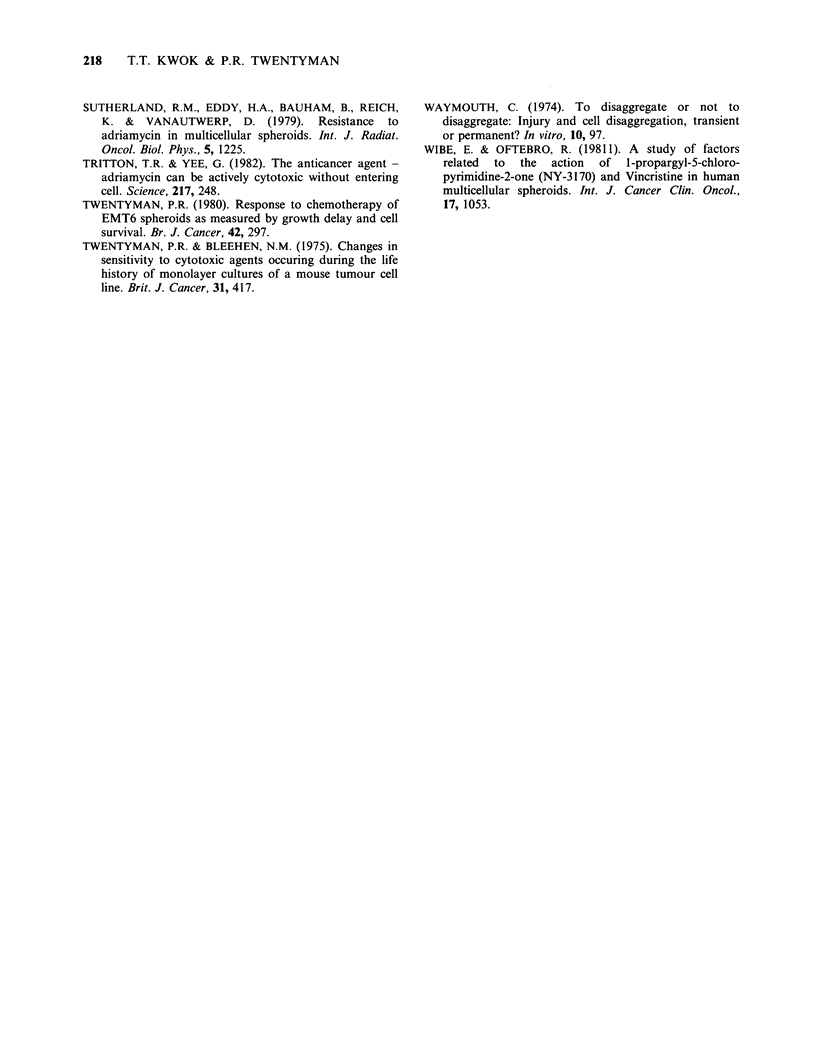# The response to cytotoxic drugs of EMT6 cells treated either as intact or disaggregated spheroids.

**DOI:** 10.1038/bjc.1985.31

**Published:** 1985-02

**Authors:** T. T. Kwok, P. R. Twentyman

## Abstract

We have compared the response to a number of cytotoxic drugs of cells treated either within intact multicellular spheroids or as isolated cells following spheroid disaggregation. The cells used were of the EMT6/Ca/VJAC mouse tumour line and spheroids were treated or disaggregated at a mean diameter of 250 micron. The response of cell to nitrogen mustard (HN2) or CCNU was similar under the two exposure conditions and we conclude that factors related to spheroid structure (i.e. drug penetrability, intercellular contact effect and microenvironment within the spheroid) do not influence the initial response to these agents. Recovery of potentially lethal damage occurring over 24 h, however, greatly modifies the level of cell killing in intact spheroids. EMT6 cells were found to be extremely resistant to vincristine under all exposure conditions. For adriamycin (ADM), cells were always initially more sensitive when exposed to the drug in suspension rather than in intact spheroids. When ADM exposure was prolonged beyond 1 h, however, delaying spheroid disaggregation for 24 h led to increased cell kill and reduced differential between the two conditions of exposure. The data suggest that both drug penetration problems and other factors related to spheroid structure are involved in determining the response of cells in small spheroids to ADM.


Br. J. Cancer (1985), 51, 211–218

### The response to cytotoxic drugs of EMT6 cells treated either as intact or disaggregated spheroids

T.T. Kwok & P.R. Twentyman

*MRC Clinical Oncology and Radiotherapeutics Unit, Hills Road, Cambridge, UK.*

**Summary** We have compared the response to a number of cytotoxic drugs of cells treated either within intact multicellular spheroids or as isolated cells following spheroid disaggregation. The cells used were of the EMT6/Ca/VJAC mouse tumour line and spheroids were treated or disaggregated at a mean diameter of 250 $\mu$m. The response of cell to nitrogen mustard (HN2) or CCNU was similar under the two exposure conditions and we conclude that factors related to spheroid structure (i.e. drug penetrability, intercellular contact effect and microenvironment within the spheroid) do not influence the initial response to these agents. Recovery of potentially lethal damage occuring over 24 h, however, greatly modifies the level of cell killing in intact spheroids. EMT6 cells were found to be extremely resistant to vincristine under all exposure conditions. For adriamycin (ADM), cells were always initially more sensitive when exposed to the drug in suspension rather than in intact spheroids. When ADM exposure was prolonged beyond 1 h, however, delaying spheroid disaggregation for 24 h led to increased cell kill and reduced differential between the two conditions of exposure. The data suggest that both drug penetration problems and other factors related to spheroid structure are involved in determining the response of cells in small spheroids to ADM.

We are currently involved in a number of investigations directed towards determining the identity and characteristics of those cells which regrow a solid tumour after chemotherapy. One aspect of these studies is the relationship between 3-dimensional tumour structure and cellular response. This relationship is being investigated using multicellular spheroids of tumour cells.

Multicellular spheroids are an *in vitro* model system showing many similarities to solid tumours *in vivo*. (Sutherland & Durand, 1976) As in the solid tumour, cells within spheroids are arranged in a 3-dimensional structure. Factors related to this structure, such as intercellular contact, may be involved in determining the response of cells within the spheroid to radiation and cytotoxic drugs. (Durand & Sutherland, 1972; Sutherland *et al.*, 1979; Deen *et al.*, 1980; Dertinger & Hülser, 1981; Wibe & Oftebro, 1981). The initial study by Durand & Sutherland (1972) showed that Chinese hamster V79 cells were more resistant to radiation when irradiated as aggregates than as single cells and these authors suggested that intercellular contact may be responsible for the observation. This concept was supported by the work of Dertinger & Hülser (1981) who showed a close relationship for a range of cell lines between relative radioresistance in the aggregated state and the extent of intercellular ionic coupling. The cells within spheroids from other cell lines have also been reported to be more resistant than single cells to treatment with hyperthermia (Durand, 1978) and cytotoxic drugs, such as adriamycin (Sutherland *et al.*, 1979), vincristine (Wibe & Oftebro, 1981) and vinblastine (Nederman, 1984), In contrast, however, single cells were found to be more sensitive than cells within intact spheroids to BCNU (Deen *et al.*, 1980) or nitrogen mustard (Hetzel & Kaufman, 1983).

In this report, we describe experiments carried out to study the relative response to a variety of cytotoxic drugs of EMT6 mouse tumour cells treated either as spheroids or as a cell suspension following spheroid disaggregation – "spheroid cells". The experiments were carried out by comparing the cell survivals (measured by clonogenic assay). In this comparison, the cell-cycle distribution is identical for both treated populations whereas this is not the case for a comparison between the cells growing as spheroids and as monolayers. However, possible effects of enzymatic spheroid disaggregation (i.e. with trypsin) on drug response have to be borne in mind using this basis of comparison. Trypsinization occurs at different times with respect to drug exposure in the protocol for intact spheroids and spheroid cells, and trypsinization is known to cause a variety of effects including membrane damage (Hebb & Chu, 1960; Waymouth, 1974). The possibility of trypsin/cytotoxic drug interaction may be a particular

Correspondence: T.T. Kwok.
Received 14 September 1984; and in revised form, 24 October 1984.

© The Macmillan Press Ltd., 1985

potential problem in the case of adriamycin for which drug the cytotoxicity has been attributed, at least in part, to membrane effects (Tritton & Yee, 1982). We have therefore carried out a number of experiments to investigate possible interactions between trypsin and cytotoxic drug damage and their implications for our comparisons. It has been previously shown that the survival of cells in drug-treated spheroids can increase if the clonogenic assay is delayed for 24h after drug treatment (Twentyman, 1980). Therefore, in our comparison between spheroid and spheroid cell response, data for the survival of cells in spheroids maintained intact for 24h after treatment have also been obtained within the same experiments.

### Materials and methods

The cells used in these studies were EMT6/Ca/VJAC mouse tumour cells and the medium was Eagle's minimal essential medium supplemented with 20% newborn calf serum (Gibco Biocult). The details of the cell line and the culture methods for spheroid growth have been described previously (Twentyman, 1980). Briefly, $5\times10^5$ cells from monolayer culture were inoculated into $75\,cm^2$ tissue culture flasks base-coated with 1% Difco Noble Agar in medium. Aggregation occurred rapidly and spheroids reached a diameter of $\sim250\,\mu m$ by Day 6.

Three flasks of spheroids of $\sim250\,\mu m$ in diameter were pooled. The spheroids were allowed to settle and the medium was discarded. The spheroids were then resuspended in 10ml medium. From this suspension, 5ml was diluted to 60ml with medium and 10ml aliquots were then transferred into plastic universal tubes for treatment of intact spheroids. The other 5ml of suspension was then used to prepare spheroid cells. The spheroids were allowed to settle and the bulk of the medium removed. Three ml of 0.075% trypsin in PBS (Gibco Biocult) was added. The spheroids were then incubated for 15min at 37°C, after which the trypsin solution was removed and 2ml of medium added. A Pasteur pipette was used to draw the spheroids up and down several times, causing them to disintegrate into single cells. The single cell suspension was made up to 60ml with fresh medium and 10ml aliquots were again transferred to plastic universal tubes for drug treatment.

### *Protocol of drug treatment*

Cytotoxic drugs were added to the tubes containing intact spheroids or spheroid cells in volume of 0.05ml to 0.2ml. Adriamycin (ADM, Farmitalia Ltd.), nitrogen mustard (HN2, Boots Co) and vincristine (VCR, Eli Lilly Ltd.) were dissolved in distilled water, and CCNU (United States N.C.I.) in absolute ethanol. These solvents alone cause no reduction in surviving fraction (Twentyman, 1980). Each tube was gassed for 5sec with a mixture of 5% $CO_2$ and 95% air, and incubated at 37°C for the appropriate period of time with continuous agitation. For CCNU and HN2, exposure of 1h was used, for VCR and ADM we used exposure times of 1, 3 and 5h. At the end of incubation, the spheroid cells were twice rinsed with 10ml fresh medium and finally resuspended in 2ml of medium. The cells were counted on a haemocytometer, appropriate dilution made and various number of cells were plated into 90mm tissue culture petri-dish (Sterilin Ltd.) containing 10ml of medium for cell-survival assay. The dishes were then incubated for 10 days at 37°C in a sealed box gas with 5% $CO_2$/95% air. The dishes were then rinsed in saline, fixed in alcohol and stained with a solution of crystal violet. Colonies of $\geqq50$ cells were counted using a binocular dissecting microscope.

Intact spheroids were also rinsed twice with 10ml fresh medium and resuspended in 2ml. From this volume, 1ml of spheroids was immediately trypsinized and disaggregated into single cell suspension for the measurement of cell survival. The remaining spheroids were transferred to a plastic universal tube coating a solid plug of 2ml 0.75% Noble Agar (Difco) and containing 9ml of fresh medium. Such tubes were incubated for 24h at 37°C in a gassing incubator before the spheroids were disaggregated and cell survival assayed as before.

### *Trypsin effect*

To study the possible influence of trypsinization on the drug response of EMT6 cells, a range of trypsin exposure time (15, 30 and 60min) was used both before drug treatment (spheroid cells) or after drug treatment (intact spheroids). Except for the time of trypsinization, the protocol was as same as that above.

### Results

Each experiment was repeated at least 3 times. In general the results of 2 experiments are shown in each Figure, the third set of data being omitted for the sake of clarity but being, in each case, similar to that shown. All the curves were fitted by eye to the data from the 2 experiments plotted.

### *Effect of trypsinization* (Figure 1)

The results of experiments to determine the effect of trypsin on the drug response of the cells in

**Figure 1** The effect of trypsinization time on the response of cells from EMT6/VJAC spheroids to 0.5 $\mu g\,ml^{-1}$ HN2 (□); 10 $\mu g\,ml^{-1}$ ADM (▲) or 5 $\mu g\,ml^{-1}$ CCNU (▽) (a) Drug exposure before trypsinization – Intact spheroid. (b) Drug exposure after trypsinization – Spheroid cell.

EMT6 spheroids are shown in Figure 1. It may be seen that for ADM (10 $\mu g\,ml^{-1}$ for 1 h) or HN2 (0.5 $\mu g\,ml^{-1}$ for 1 h) the measured cell survival did not depend upon the length of the trypsin exposure time for trypsinization either before or after drug exposure. For CCNU, there was a tendency for the measured cell survival to decrease as the time of exposure to trypsin increases. The magnitude of the decrease was similar for trypsinization either before or after CCNU exposure. In all experiments, trypsin exposure of up to 1 h had no effect on the viability of control cells and the plating efficiency remained within the range 60–100%

*Vincristine*

In four experiments using VCR exposure of 1 $\mu g\,ml^{-1}$ for 1 h, the mean surviving fractions obtained were 0.73 (spheroid cells) and 0.83 (intact spheroids). In two experiments using higher doses of VCR, the mean surviving fractions for 5 $\mu g\,ml^{-1}$ were 0.78 (spheroid cells) and 0.80 (intact spheroids) and for 10 $\mu g\,ml^{-1}$ the surviving fractions were 0.70 (spheroid cells) and 0.69 (intact spheroids). With an extended exposure time of 5 h, survivals following 2 $\mu g\,ml^{-1}$ VCR were 0.73 (spheroid cells) and 0.85 (intact spheroids).

*Nitrogen mustard* (Figure 2)

The dose response curve for spheroid cells was very close to that of intact spheroids with immediate disaggregation. The curve for intact spheroids with 24 h dissaggregation delay showed a marked reduction in its slope.

*CCNU* (Figure 3)

The results were very similar to that for HN2. The intact spheroids (immediate disaggregation) and spheroid cells showed a similar dose-response pattern while the curve for intact spheroids with 24 h delay was less steep.

*Adriamycin* (Figure 4)

The dose response curves for spheroid cells and for intact spheroids (either immediate or delayed disaggregation) incubated with ADM for 1 h

**Figure 2** Response to nitrogen mustard (HN2) of isolated spheroid cells (●), and cells from intact spheroids with immediate disaggregation (■) and 24 h delayed disaggregation (▲).

**Figure 3** Response to CCNU of isolated spheroid cells (●), and cells from intact spheroids with immediate disaggregation (■) and 24 h delayed disaggregation (▲).

**Figure 4** Response to adriamycin (ADM) of isolated spheroid cells (●), and cells from intact spheroids with immediate disaggregation (■) and 24 h delayed disaggregation (▲). Exposure times, 1 h (a); 3 h (b) and 5 h (c).

(Figure 4a), 3 h (Figure 4b) or 5 h (Figure 4c) were biphasic with an inflexion point at $\sim 5 \mu g ml^{-1}$. Beyond this dose, only relatively small additional increases in cell killing were seen. For 1 h exposure (Figure 4a), the sensitivity of spheroid cells is greater than that of cells in intact spheroids. There is no apparent difference for intact spheroids between immediate and delayed disaggregation. For 3 h exposure (Figure 4b), spheroid cells were again more sensitive than intact spheroids (immediate disaggregation) but intact spheroids (delayed disaggregation) had an increased sensitivity similar to that of spheroid cells. For 5 h exposure (Figure 4c), the sensitivity of these 3 groups is in the order spheroid cells > intact spheroids (delayed disaggregation) > intact spheroids (immediate disaggregation).

### Discussion

*Effect of trypsinization*

The aim of this report was to compare the response of isolated spheroid cells and cells in intact spheroid to 4 different cytotoxic drugs, ADM, VCR, HN2 and CCNU. The process of trypsinization took place before the drug treatment for isolated spheroid cells but after drug exposure for intact spheroids. It is necessary, therefore, to know whether or not the process of trypsinization affects the drug response. From the data shown in Figure 1, it is seen that changing the length of the trypsinization period from 15 min to 1 h (before or after the drug exposure) does not change the sensitivity of the cells from spheroids to ADM or HN2. It would therefore, seem resonable to assume that our results are unlikely to be affected by the temporal relationship of the standard 15 min trypsin exposure period to the drug treatment. For CCNU, as the time of trypsinization increases, the apparent cell killing by CCNU increases. The two curves (i.e. trypsinization before or after exposure) have similar slopes. Therefore, while there may be some interaction between trypsin damage and CCNU cytotoxicity, it is unlikely that a comparison of before or after trypsinization for drug exposure will be significantly affected by this factor.

*Cytotoxic drug responses*

In addition to the inherent sensitivity of the tumour cells, a number of factors may influence the lethal action of a particular drug on the cells in multicellular spheroids. These include: 1. Drug penetration; 2. Cell cycle distribution; 3. Intercellular contact effect and 4. Microenvironment (i.e. gradients of oxygen, glucose, pH, etc.) of the cells within the spheroid. All these factors are related to the 3-dimensional structure of the spheroid. Since in this report the drug responses of isolated spheroid cells and cells within spheroids are compared, the cell cycle distribution of both treated populations will be the same and need not be considered further. The spheroids used in the present study were $\sim 250 \mu m$ in diameter, i.e. $\sim$9–10 cell layers from centre to periphery. Although such spheroids do not have a necrotic central region, there is no doubt that gradients of oxygen, glucose, etc. will be present. These microenvironmental factors together with possible drug diffusion and influence of intercellular contact may be considered collectively as "spheroid structural factors" (SSFs). The intercellular contact effect, according to Durand *et al.* (1972) can be sub-divided into 2 categories, "inherent" and "non-inherent". The "non-inherent" part is absolutely dependent on the intact structure of the spheroid at the time of radiation. Once this structure is destroyed, it will no longer exist, while the "inherent" part will still be "remembered" by the spheroid cells and this memory will gradually decay over a period of hours. In this report, spheroid cells were exposed to the drugs immediately after they were disaggregated from the spheroids. Therefore, it should be kept in mind that the conclusion made is only on the "non-inherent" but not the "inherent" intercellular contact effect.

EMT6 cells appeared to be very resistant to VCR as the survival from intact spheroid or spheroid cell was always greater than 0.7 even when the exposure time was 5 h and the dose was $2 \mu g ml^{-1}$. It has been shown that the resistance of cells in spheroids to VCR may be partly related to the limited penetration of this drug in the spheroid and partly to the "out of cycle" state of many cells in spheroids (Wibe & Oftebro, 1981). Limited penetration into spheroids has also been shown for a closely-related drug, vinblastine (Nederman, 1984). However, we have been able to show a marked resistance to VCR of EMT6/Ca/VJAC cells in both exponential and plateau phase monolayer growth (unpublished data) and hence it appears that EMT6 cells have a high intrinsic resistance to this drug and no other factors need to be involved in the spheroid cell resistance observations.

The data presented in Figures 2 and 3 show that dose-response curves of cells in intact spheroids and isolated spheroid cells to CCNU or HN2 are very similar. It therefore appears that SSFs have little or no influence on the response of EMT6 cells to CCNU and HN2. These results are in contrast to the results from the spheroids of 9L rat brain tumour cells (Deen *et al.*, 1980) and V79 cells (Hetzel & Kaufman, 1983) which showed single cells to be more resistant than cells in spheroids. The study of Hetzel and Kaufman using HN2 was a comparison of cells in spheroids with cells in monolayer (both exponential and plateau phase) and the cell cycle distribution of the treated population was different. Unlike EMT6 cells which are more sensitive to HN2 in exponential than in plateau phase (Twentyman & Bleehen, 1975), V79 cells used by Hetzel & Kaufman (1983) were more sensitive whilst in plateau phase. The big difference in sensitivity of cells in spheroids compared with exponential phase cells may be largely attributed therefore to the "out of cycle" nature of many of the cells in spheroids. The additional sensitivity of spheroid cells over and above plateau phase cells, however, must have been due to some other factors related to spheroid structure. It is clear from our results that such a factor does not operate in the type of comparison which we have made. Similarly, the results obtained by Deen *et al.* (1980) for BCNU in 9L spheroids using a comparison closely similar to that which we have used seem likely to be governed by some factors related to spheroid structure. One cannot rule out however the possibility of disaggregation artefacts being involved in their results. Once again, however, no such factors appear to be involved in determining the response of EMT6 spheroids to CCNU.

In addition to possible differences in initial sensitivity of isolated spheriod cells and cells in intact spheroids to drugs, an additional factor comes into operation when considering the relationship between cellular response and the "overall' response of intact spheroids. "Recovery of potentially lethal damage" by cells in drug-treated EMT6 spheroids was reported by Twentyman (1980). It would have been interesting to examine whether or not such a recovery also occurs in spheroid cells treated with drugs after disaggregation and held as a single cell suspension in conditions not conductive to cell cycling. Because of the very high propensity of re-aggregation in EMT6 suspensions, however, it was not possible to carry out such experiments. Whether or not recovery from "potentially lethal damage" is directly dependent upon intercellular contact or microenvironment during the post-treatment period remains, therefore, a matter of speculation.

Adriamycin, although showing activity against quite a wide spectrum of solid tumours *in vivo,* has limited penetrability in spheroids (Sutherland *et al.*, 1979; Durand, 1981). In order to separate the drug penetration effect from other SSFs in determining the response of EMT6/VJAC spheroids, the cell survivals for 2 and 10 $\mu g\,ml^{-1}$ and 1, 3 and 5 h exposure to ADM (from Figure 4) have been re-plotted in Figure 5. For spheroid cells, the curve of

**Figure 5** Changes in cell survival with exposure time to ADM of isolated spheroid cells (●, ○) and cells from intact spheroid with immediate disaggregation (■, □). Open symbols = 2 $\mu g\,ml^{-1}$. Closed symbols = 10 $\mu g\,ml^{-1}$.

surviving fraction *versus* exposure time is linear for 2 $\mu g\,ml^{-1}$ and convex upwards for 10 $\mu g\,ml^{-1}$. For cells treated in intact spheroids, however, both curves are concave upwards. The sensitivity of isolated spheroid cells is always greater than that of cells in intact spheroids but the differential increases with increasing time of drug exposure. This is the opposite to which would be expected if penetrability were the only factor involved. If this were the case, as increasing exposure time allowed the drug concentration at the centre of the spheroid to approach more closely the concentration in the medium, the curves would be expected to converge. We have observed that after exposure to 15 $\mu g\,ml^{-1}$ of ADM for 3 h, the outer 8–9 layers of cells in large EMT6/VJAC spheroids (~800 $\mu m$ in diameter) are brightly fluorescent (unpublished data). Therefore, for the small spheroids (9–10 cell layer from centre to periphery) used in the present study, 3 h or even longer exposure time should allow penetration of ADM to all cells in the spheroids. It would appear therefore that some aspect of SSFs may also be involved in resistance of cells in intact spheroids to ADM when compared with isolated spheroid cells. Similar conclusions were reached in the EMT6/Ro spheroid (Sutherland *et al.*, 1979) and the V79 spheroid (Durand, 1981). The influence of "time of clonogenic assay" upon the measured survival of cells treated in intact spheroids is dependent upon the exposure time to the drugs. The dose-response curves for 1 h exposure to ADM (Figure 4a) are very similar for immediate disaggregation and for 24 h delay. If the exposure time is >1 h, as shown in Figures 4b and 4c, the measured cell killing by ADM is much greater if the clonogenic assay is delayed. Also, for 3 and 5 h exposure, the ratio of survivals measured at the two times (24 h compared with 0 h) decreases as the dose increases and the pattern for both 3 and 5 h exposures are very similar (Figure 6). Thus, the enhancement effect by delay of cologenic assay may be dependent upon both drug dose and exposure time. In an *in vivo* study, ADM was shown to have a longer half-life in solid EMT6 tumours (~67 h) than in other normal mouse tissues (Siemann & Sutherland, 1979). Also, after a single i.v. injection of ADM, the plasma ADM half-life between 15 min and 8 h was ~2 h (Rosso *et al.*, 1973). Therefore, the results from the spheroids with delayed disaggregation may be more relevant to the *in vivo* data than those with immediate disaggregation. Also, this finding should explain why ADM is low in penetrability for short exposure but clinically quite active in a wide range of solid tumours.

**Figure 6** Relative survivals (disaggregation at 24 h/ disaggregation at 0 h) of cells from EMT6 spheroids after 1 h (△), 3 h (■) or 5 h (▼) exposure to graded dose of ADM. All the points were taken from the data in **Figure 4**.

We thank Ms. Karen Wright for providing a constant supply of EMT6 cells and Prof. N.M. Bleehen for his interest and encouragement.

### References

DEEN, D.F., HOSHINO, T., WILLIAMS, M.E., MURAOKA, I., KNEBEL, K.D. & BARKER, M. (1980). Development of a 9L rat brain tumour cell multicellular spheroid system and its responses to 1,3-bis(2-chloroethyl)-1-nitrosourea and Radiation. *J. Natl Cancer Inst.*, **64**, 1373.

DERTINGER, H. & HÜLSER, D. (1981). Increased radioresistances of cell in cultured multicell spheroids. I. Dependence on cellular interaction. *Radiat. Environ. Biophys.*, **19**, 101.

DURAND, R.E. (1978). Effect of hyperthermia on the cycling, non-cycling and hypoxic cells or irradiated and unirradiated multicell spheroids. *Radiat. Res.*, **75**, 373.

DURAND, R.E. (1981). Flow cytometry studies of intracellular adriamycin in multicell spheroids *in vitro*. *Cancer Res.*, **41**, 3495.

DURAND, R.E. & SUTHERLAND, R.M. (1972). Effect of intercellular contact on repair of radiation damage. *Exp. Cell Res.*, **71**, 75.

HEBB, C.R. & CHU, M.-Y.W. (1960). Reversible injury of L-strain mouse cells by trypsin. *Exp. Cell. Res.*, **20**, 453.

HETZEL, F.W. & KAUFMAN, N. (1983). Chemotherapeutic drugs as indirect oxygen radiosensitizers. *Int. J. Radiat. Oncol. Biol. Phys.*, **9**, 751.

NEDERMAN, T. (1984). Effect of vinblastine and 5-fluorouracil on human glioma and thyroid cancer cell monolayers and spheroids. *Cancer Res.*, **44**, 254.

ROSSO, R., ESPOSITO, M., SALA, R. & SANTI, L. (1973). Distribution of daunomycin and adriamycin in mice. A comparative study. *Biomedicine*, **19**, 304.

SIEMANN, D.W. & SUTHERLAND, R.M. (1979). A comparison on the pharmacokinetics of multiple and single dose administration of adriamycin. *Int. J. Radiat. Oncol. Biol. Phys.*, **5**, 1271.

SUTHERLAND, R.M. & DURAND, R.E. (1976). Radiation response of multicell spheroids – An *in vitro* tumour model. Current topics in *Radiat. Res. Quart.*, **11**, 87.

SUTHERLAND, R.M., EDDY, H.A., BAUHAM, B., REICH, K. & VANAUTWERP, D. (1979). Resistance to adriamycin in multicellular spheroids. *Int. J. Radiat. Oncol. Biol. Phys.*, **5,** 1225.

TRITTON, T.R. & YEE, G. (1982). The anticancer agent – adriamycin can be actively cytotoxic without entering cell. *Science*, **217,** 248.

TWENTYMAN, P.R. (1980). Response to chemotherapy of EMT6 spheroids as measured by growth delay and cell survival. *Br. J. Cancer*, **42,** 297.

TWENTYMAN, P.R. & BLEEHEN, N.M. (1975). Changes in sensitivity to cytotoxic agents occuring during the life history of monolayer cultures of a mouse tumour cell line. *Brit. J. Cancer*, **31,** 417.

WAYMOUTH, C. (1974). To disaggregate or not to disaggregate: Injury and cell disaggregation, transient or permanent? *In vitro*, **10,** 97.

WIBE, E. & OFTEBRO, R. (19811). A study of factors related to the action of 1-propargyl-5-chloro-pyrimidine-2-one (NY-3170) and Vincristine in human multicellular spheroids. *Int. J. Cancer Clin. Oncol.*, **17,** 1053.